# Incidence and medical management of bisphosphonate-associated atypical femoral fractures in a major trauma centre: a retrospective observational study

**DOI:** 10.1186/s12891-017-1392-9

**Published:** 2017-01-23

**Authors:** Neil Eisenstein, Ganesh Kasavkar, Dhruva Bhavsar, Faisal Shehzaad Khan, Zoe Paskins

**Affiliations:** 1 Royal Centre for Defence Medicine, Birmingham, UK; 2 Department of Trauma and Orthopaedics, Royal Stoke University Hospital, Stoke-On-Trent, UK; 3 Department of Rheumatology, Haywood Hospital, Stoke-On-Trent, UK; 4 Research Institute for Primary Care & Health Sciences, Keele University, Keele, ST5 5BG UK; 5 School of Chemical Engineering, University of Birmingham, Edgbaston, Birmingham, B15 2TT UK

**Keywords:** Atypical, Fracture, Bisphosphonate, Denosumab, Femur, Subtrochanteric, Osteoporosis

## Abstract

**Background:**

Atypical femoral fractures (AFFs) are rare events associated with increased duration of bisphosphonate exposure. Recommended management of AFFs include cessation of bisphosphonates and imaging of the contralateral femur. The aims of this study were to identify the local incidence of AFFs in bisphosphonate users and to audit the medical management of AFFs against published recommendations.

**Methods:**

A retrospective analysis of the admissions database for a major trauma centre identified all femoral fractures (3150) in a five-year period (July 2009 to June 2014). Electronic health records and radiographs were reviewed using the 2013 American Society for Bone and Mineral Research (ASBMR) diagnostic criteria for AFF to establish the number of cases. To estimate incidence, the total number of bisphosphonate users was derived from primary care prescription and secondary care day-case records. Medical management of cases with AFF on bisphosphonates was audited against guidance from ASBMR and Medicines & Healthcare Products Regulatory Agency.

**Results:**

10 out of 3150 femoral fractures met criteria for AFF; 7 of these patients had a history of exposure to bisphosphonates (6 oral, 1 intravenous). There were 19.1 AFFs per 100,000 years of bisphosphonate use in our region. Bisphosphonates were stopped and the contralateral femur imaged in only 2 of the 7 patients treated with bisphosphonates.

**Conclusion:**

Our local incidence is in line with published figures; however, this is the first published evidence suggesting that medical management and identification of AFF may be suboptimal. Managing these patients remains challenging due to their rarity and possible lack of awareness.

## Background

Atypical femoral fractures (AFFs) are rare injuries that have received increasing attention in the scientific literature in recent years. There is growing evidence that bisphosphonate exposure contributes to the risk of these fractures. AFFs have also been reported after treatment with other, non-bisphosphonate, antiresorptives such as Denosumab [1]. In 2013, the American Society for Bone and Mineral Research (ASBMR) task force published their second report on AFFs in which the case definition was refined in light of new evidence [2]. In summary, the case defining criteria may be divided into clinical, anatomical, and radiographic categories. These fractures occur through low energy mechanisms. They are located between the lesser trochanter and the supracondylar flare. They are non- or minimally comminuted and originate from the lateral cortex with evidence of cortical flaring or beaking. Specific exclusions include neck of femur, periprosthetic, and pathological fractures.

Subsequent to the publication of the first report of the ASBMR taskforce, and the adoption of the 2010 case-defining criteria [3], several studies attempted to investigate the epidemiology of AFFs in the USA, Switzerland, and Sweden. The heterogeneous methodology employed has made comparisons challenging. However, studies using radiographic adjudication against ASBMR criteria indicate the incidence of AFF varies from 1.8 to 113 per 100,000 patient years of exposure to bisphosphonates [4–7]. A more recent study, using the 2013 ASBMR criteria, identified an incidence of 110 per 100,000 patient years of exposure [8].

The relationship between bisphosphonate exposure and AFF is thought to be due to the inhibition of osteoclastic activity with a coupled inhibition of osteoblasts leading to failure of repair of microfractures [4, 8, 9]. Unlike osteonecrosis of the jaw (ONJ), which is most commonly seen in patients on high-dose bisphosphonates for malignancy [10], AFFs are most likely to occur in patients on standard-dose treatment, although a dose-response relationship has been identified [9]. Further, while they were first described in a small group of patients treated with alendronate [11], the contribution of bisphosphonates seems to be a class effect rather than specific to a single drug [2]. Concern about atypical fractures underpins the increasing move to promote bisphosphonate drug holidays; however, it is estimated that for every AFF that develops, 36 insufficiency fractures are prevented [12].

Both the ASBMR [2, 3] and MHRA [13] provide guidance on the medical management of AFFs which includes: cessation of antiresorptive; consideration of teriparatide in cases of poor fracture healing; ensuring the patient is replete in calcium and vitamin D, and ensuring the contralateral femur is imaged. In the absence of randomised controlled trials, this is based on expert opinion and anecdotal case reports. In terms of operative treatment, the 2013 ASBMR report recommends the use of intramedullary nailing and the avoidance of locking plates.

The aims of this study were to identify the number of cases of AFF in our centre, calculate incidence of AFF in bisphosphonate users, and to audit the medical management of AFF.

## Methods

A retrospective review of an electronic trauma admissions database at a major trauma centre was performed. Records were retrieved for a 5-year period from 01/07/2009 to 30/06/2014. Initially, all femoral fractures were identified; subsequently, information in the database allowed the exclusion of neck of femur fractures, high-energy injuries, pathological fractures, and peri-prosthetic fractures. The definition of pathological fractures used in this study is taken from the ASBMR report: “fractures associated with primary or metastatic bone tumors and miscellaneous bone diseases (e.g. Paget’s disease, fibrous dysplasia).”

The radiographs of all of the remaining femoral fractures were reviewed by one author (NE) to determine whether they met the radiological and anatomical inclusion criteria: location from the lesser trochanter to the flair of the femoral condyle, origination at the lateral cortex, cortical beaking, periosteal thickening, and substantially horizontal fracture orientation. A random sample of 50 out of those selected for radiograph review were reviewed independently by both ZP and NE in order to establish inter-rater agreement against inclusion criteria. At the time of radiographic adjudication the authors were blinded to the exposure of the patients to antiresorptive treatment. Electronic health records of the confirmed atypical femoral fracture group were reviewed to determine demographic information, bisphosphonate exposure, calcium and vitamin D use, glucocorticoid and proton pump inhibitor use and management.

To determine the number of patient years of bisphosphonate treatment over the audit period, the total number of oral bisphosphonate prescriptions issued in primary care over the audit period was identified from data analysts in two local Clinical Commissioning Groups that cover the catchment area of the acute trust. The footprint of these CCGs maps directly to the catchment of the acute trust and all patients with femur fractures in these catchment areas would automatically be referred to this trauma centre for fracture repair, and not elsewhere. In addition, data on the number of intravenous bisphosphonate users over the same period was estimated based on the number receiving treatment in 2015. These figures were combined to calculate the total number of patient years of BP exposure over the 5 year period.

Medical management of bisphosphonate-associated AFF patients was audited against the following standards:

- cessation of bisphosphonate treatment [2]
- assessment of calcium and vitamin D status with supplementation if required [2]
- radiographic examination of the contralateral femur [13]

Assessment of calcium and vitamin D status was evaluated by looking for comments on calcium intake or vitamin D status in the electronic health records, prescription of calcium and vitamin D or serum measurement of vitamin D. As the primary aim of the study was to audit medical management, ethical approval was not deemed necessary.

## Results

**Fig. 1:**
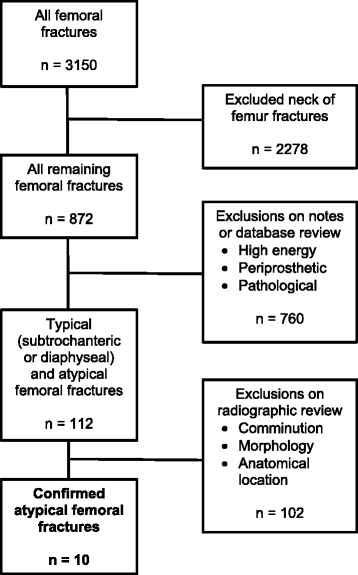
Flow chart demonstrating case identification process. This flowchart outlines the steps taken to identify cases of AFF as defined by the 2013 ASBMR Report. Clinical and radiological inclusion and exclusion criteria are described in greater detail in the methods section

Figure 1 demonstrates the process used to identify the definitive cases of atypical femoral fracture. Of 3150 femoral fractures, 112 were selected for radiograph review. 10 fractures were confirmed AFF over the 5 year period, representing 0.32% (10/3150) of all femoral fractures (including neck of femur fractures) and 8.9% (10/112) of all low energy femoral shaft and subtrochanteric fractures. There was complete agreement between the two adjudicators for radiographic diagnosis of AFF against ASBMR 2013 criteria (Cohen’s Kappa statistic of 1).

Seven of the 10 patients with confirmed AFF had previously been exposed to bisphosphonates: 6 oral alendronic acid and 1 intravenous Pamidronate. One additional patient who met the radiographic criteria had pre-fracture exposure to Denosumab but also had chronic kidney disease stage 5 and was therefore excluded due to the high possibility of renal metabolic bone disease. One patient, not on bisphosphonates, was treated with oral glucocorticoids. Three bisphosphonate treated patients were also treated with proton pump inhibitors. No patients had a diagnosis of rheumatoid arthritis.

The mean age of the AFF cohort was 72.8 years (range: 57 to 88) and all but one of the patients were female. The duration of bisphosphonate use ranged from 3–15 years (it was not possible to obtain these data for 2 patients). See Table 1 for the demographic data for each patient.

**Table 1:** Atypical Femoral Fracture cases demographics and medical management

Sex	Age	Radiographic features of AFF^a^	Antiresorptive treatment pre-fracture	Cessation of BP post fracture^b^	Contralateral imaging performed	Assessment of Calcium and Vitamin D status^c^	Operative Management^d^
F	66	All major criteria 1 minor	None	N/A	No	No	Proximal femoral locking plate
M	77	All major criteria 1 minor	Alendronic Acid	No	No	No	IM Nail
F	88	All major criteria^e^ 1 minor	None	N/A	No	No	DHS
F	80	All major criteria 1 minor	None	N/A	No	No	IM Nail
F	63	All major criteria^f^	Alendronic Acid	No	No^f^	No	IM Nail
F	69	All major criteria 1 minor	Alendronic Acid	Yes	Yes	No	Bilateral IM Nail
F	77	All major criteria 1 minor	Pamidronate	No	No	on supplements	IM Nail
F	70	All major criteria 1 minor	Alendronic Acid	No	No	on supplements	IM Nail
F	85	All major criteria 1 minor	Alendronic Acid	Yes	Yes	on supplements	IM Nail
F	57	All major criteria 1 minor	Alendronic Acid	No	No	No	IM Nail

^a^ It was not possible to determine the minor features relating to prior symptoms, or to determine bilaterality when contralateral imaging was not performed. When a minor feature was present, this related to increased cortical thickening. Poor healing was not mentioned in any electronic health records

^b^ N/A not applicable

^c^ Determined by mention of calcium and vitamin D status in the electronic health record, check of serum vitamin D or whether patient was on supplements

^d^ IM intramedullary nail; DHS dynamic hip screw

^e^ This fracture was incomplete

^f^ This patient had sustained a midshaft fracture on the contralateral side 3 years previously

Primary care records identified 34,157 patient-years of oral bisphosphonate treatment prescribed in the region during the study period. Additionally, 2485 patient-years of exposure to intravenous bisphosphonates were estimated from secondary care day-case records. 7 AFFs occurred in 36642 patient years of treatment equating to an estimated incidence of 19.1 per 100,000 patient years of bisphosphonate exposure. In terms of operative management, 8 patients with AFF were treated with intramedullary nailing (of which one patient also received prophylactic nailing of the contralateral femur), 1 was treated with a dynamic hip screw and 1 was treated with a proximal locking plate.

Medical and operative management is summarised in Table 1. Of the 7 patients with pre-fracture exposure to bisphosphonates, 2 had bisphosphonates discontinued. In these two patients, the treating orthopaedic surgeon had diagnosed AFF. A diagnosis of AFF was not made in the other 5 cases by the treating orthopaedic surgeon. Eight of the 10 radiographs were reported and none of the radiologists’ reports mentioned AFF. Imaging of the contralateral femur was performed in 2 of the 7 confirmed AFF cases on bisphosphonates. One of these had increased cortical thickening on the contralateral side and proceeded to have prophylactic nailing. No patients had bilateral AFF at the time of the index event although one patient had a midshaft fracture on the contralateral side 3 years prior to the AFF identified in this study (pre-fixation radiographs of the contralateral fracture not available).

Out of the 10 confirmed AFF patients, 3 patients were discharged on calcium/vitamin D supplements. In the other 7 patients, there was no mention in the electronic health record of assessment of calcium and vitamin D status and serum vitamin D was not checked at the time of fracture. Orthopaedic follow up was insufficiently long to judge healing in 5 of the 7 bisphosphonate related AFF, however no patient represented with symptomatic delayed or non-union to date. Of the 2 patients with formal follow up of more than 9 months no concerns were recorded relating to fracture healing. Subsequent to this study, all patients’ general practitioners were contacted to inform them of the diagnosis of AFF, suggest any BP be discontinued and invite the patients for review in a secondary care bone clinic to review osteoporosis management.

## Discussion

This study has demonstrated that, using the updated ASBMR 2013 criteria, there were 10 AFFs diagnosed at our Major Trauma Centre in the 5 years from July 2009 to June 2014. Of these, 7 were associated with bisphosphonate use. This is the first study to estimate the incidence of AFF in the UK using the updated ASBMR criteria. The local estimated incidence of 19.1 AFFs per 100,000 years of bisphosphonate treatment is similar to previous radiographically adjudicated studies using either the 2010 or 2013 ASBMR case definitions of AFF [5, 7]. However the estimated incidence in this study and others is markedly lower than that found by investigators in Sweden who report rates of 5–11 per 10,000 years of bisphosphonate use [4, 8].

In addition, this is the first published audit of the medical management of these rare events. Our results show that several patients did not receive optimal post-AFF medical management as described in the guidance published by the MHRA and ASBMR. One possible explanation for this finding is that these fractures were diagnosed correctly but there was a subsequent lack of awareness of recommended management guidelines. However, it is the conjecture of the authors that some of these injuries were not recognised for what they were at the time of hospitalisation by orthopaedic surgeons or radiologists. This is the first study to our knowledge that has reported on whether or not AFF was diagnosed at the time of event by orthopaedic surgeon; however a previously published Canadian audit demonstrated that AFFs were not recognised by radiologists in all but one of 24 cases [14].

The reason for this is likely to be the infrequency of clinicians’ exposure to AFFs. While the exact number varies over time, our Major Trauma Centre has approximately 12 consultants admitting trauma. This means that, on average, each consultant might be expected to admit an AFF only once every 6 years (or only every 8.6 years for an AFF in a patient exposed to bisphosphonates).

The calculation of incidence is subject to three important limitations. First, our estimate of the number of years of oral bisphosphonate exposure is calculated using prescription data. Adherence to bisphosphonate treatment is poor with as many as half of patients discontinuing at one year [15]. As a result, the number of years of actual bisphosphonate exposure may be inflated and the incidence underestimated. Additionally, an approximation of the number of patient-years of intravenous bisphosphonate therapy was made due to limitations of secondary care prescribing records. As there are proportionately far fewer patients on injectable bisphosphonates than oral treatment, (6.8% of the total) we do not believe this estimation will have had a marked effect on our results. Finally, we have made an assumption that the catchment of the CCGs matched the acute Trust catchment. In 2012, the Trust was awarded major trauma status to act as a tertiary centre for the wider population of 2.25 million in the North West Midlands and North Wales; as AFFs are not a product of major trauma, we considered this not to have adversely affected our results. The addresses of all the bisphosphonate treated AFF cases were confirmed as belonging to our CCG catchment areas. It is possible the hospital treated patients with fracture from neighbouring CCGs, which would result in an overestimate of incidence.

This study has three important messages for clinical practice. First, we have identified that patients with AFF are at risk of being managed without reference to the recommendations of the ASBMR or MHRA. By publishing our data, we hope to raise awareness of these rare events and their management. Previous reports suggest between 22 and 83% of those with AFF have a bilateral fracture or radiographic abnormality and therefore contralateral imaging is crucial. The authors acknowledge, however, that the published management recommendations based on drug cessation are based on low-level evidence. This is due to the difficulty in powering high quality studies that investigate such rare events. One possible solution to both raise awareness within orthopaedics and to investigate association and outcomes might be to establish a national or international registry of these fractures.

The second important clinical message from these findings is that despite being rare overall, these injuries make up nearly 9% of all “low energy” femoral fractures. Therefore, a diagnosis of “low-energy” femoral fracture should prompt a high level of suspicion of AFF. Finally, robust local pathways need to be developed between admitting teams and osteoporosis services so that, when AFFs are recognised, the patients’ osteoporosis treatment may be reviewed appropriately.

## Conclusion

AFFs are rare and can occur both with and without exposure to bisphosphonates. Our local incidence is comparable to internationally reported figures. Medical management is challenging due to the rarity of these events. We propose that increased awareness and multi-speciality working would serve to improve the management of these patients.

## Abbreviations

AFF: Atypical femoral fracture
ASBMR: American Society for Bone and Mineral Research
DHS: Dynamic hip screw
IM: Intra-medullary
MHRA: Medicines and Healthcare products Regulatory Authority
